# Experience of stigma and discrimination and the implications for healthcare seeking behavior among people living with HIV/AIDS in resource-limited setting

**DOI:** 10.1080/17290376.2013.806645

**Published:** 2013-05-30

**Authors:** Taddese Alemu, Sibhatu Biadgilign, Kebede Deribe, Horacio Ruiseñor Escudero

**Keywords:** HIV/AIDS, stigma, discrimination, EPPM, Ethiopia, VIH/SIDA, la stigmatisation, la discrimination, EPPM, Ethiopie

## Abstract

**Background:**

Stigma and discrimination can limit access to care and treatment services. Stigma hides HIV from the public, resulting in reduced pressure for behavioral change. For effective behavior change, empirically grounded and theory-based behavioral change approaches are fundamental as a prevention interventions directed on decreasing stigma and discrimination. The objective of the study was to assess the experience of stigma and discrimination on the psychosocial and health care seeking behavior of people living with HIV/AIDS (PLHIV) in Arba Minch, Ethiopia.

**Methods:**

This study uses qualitative methods involving focus-group discussions and in-depth interviews conducted in Arba Minch town and nearby Kebeles. Our sample consisted of PLHIV and other key informants who were purposively selected. Data were analyzed manually using thematic content analysis framework.

**Results:**

It appears that the magnitude of stigma and discrimination in the area has decreased to a considerably lower level, however, the problem's severity is still being influenced by various factors including: current residence, disclosure status and level of community's awareness about HIV/AIDS. Care and support services provided to PLHIV were well accepted by the respondents and the majority of them were willing to make use of any service available. Health information messages that have been disseminated to the public through mass media since the start of the epidemic in 1984 and AIDS cases in 1986 have played a significant role regarding the current prevailing problem of stigma and discrimination of PLHIV.

**Conclusion:**

Stigma and discrimination have come to a level that can be tolerated by most PLHIV that live in this region, especially those who have disclosed their HIV status and were living in urban areas. This calls for a strategy that improves the rates of serostatus disclosure after HIV counseling and testing and strengthens and integrates activities in the task of expanding care and support activities.

## Abstract (FR)

**Contexte:**

La stigmatisation et la discrimination peuvent limiter l'accés aux soins et aux services de traitement. La stigmatisation cache le VIH du public, ce qui entraîne une pression réduite pour un changement de comportement. Pour un changement de comportement efficace, des approches de changement de comportement empiriquement fondées et basées sur la théorie sont fondamentales comme des interventions de prévention visant à diminuer la stigmatisation et la discrimination. L'objectif de l'étude était d'évaluer l'expérience de la stigmatisation et de la discrimination sur le comportement de recherche psychosociale et de soins de la santé des personnes vivant avec le VIH/sida (PVVIH) à Arba Minch, Ethiopie.

**Méthodes:**

Cette étude utilise des méthodes qualitatives impliquant des discussions de groupe et des entrevues en profondeur menées dans la ville d'Arba Minch et Kebeles, à proximité. Notre échantillon était composé de PVVIH et d'autres informateurs clés qui ont été choisis à dessein. Les données ont été analysées manuellement à l'aide du cadre d'analyse thématique de contenu.

**Résultats:**

Il semble que l'ampleur de la stigmatisation et de la discrimination dans la région a diminué à un niveau nettement inférieur, cependant, la gravité du problème est toujours influencée par divers facteurs, notamment: la résidence actuelle, le statut de la divulgation et le niveau de sensibilisation de la communauté au sujet du VIH/SIDA. Les services de soins et de soutien offerts aux PVVIH ont été bien acceptés par les répondants et la majorité d'entre eux étaient prêts à profiter de tous les services disponibles. Les messages d'information sur la santé qui ont été diffusées auprès du public à travers les médias depuis le début des cas épidémiques en 1984 et le sida en 1986 ont joué un rôle important en ce qui concerne le problème courant qui prévaut de la stigmatisation et la discrimination des PVVIH.

**Conclusion:**

La stigmatisation et la discrimination est venu à un niveau qui peut être toléré par la plupart des PVVIH qui vivent dans cette région, en particulier ceux qui ont divulgué leur séropositivité et vivaient dans des zones urbaines. Ceci appelle à une stratégie qui améliore les taux de divulgation de séropositivité après la consultation et dépistage du VIH (CDV) ainsi que renforce et intègre les activités dans la tâche de développement des soins et du soutien.

## Background

The 2010 UNAIDS report estimates that 33.3 million (31.4–35.3 million) people were living with HIV, with Sub-Saharan Africa still bearing a substantial share of the global HIV burden with 22.5 million (20.9–24.2 million) people living with HIV/AIDS (PLHIV), representing 68% of the global HIV burden. Some of largest epidemics in Sub-Saharan Africa—Ethiopia, Nigeria, South Africa, Zambia and Zimbabwe—have either stabilized or are showing signs of decline (UNAID/WHO 2010). Since Ethiopia reported its first HIV case in 1984 and its first AIDS cases in 1986, the HIV/AIDS epidemic has evolved into a generalized epidemic (Disease Prevention and Control Department 2005). Evidence suggests that the stigma associated with HIV/AIDS has a negative effect over one's decision to get tested for HIV, to obtain adequate health care and on the proportion of PLHIV that disclose their serostatus to a sex partner (Golin, Isasi, Bontempi & Eng 2002; Herek, Mitnick, Burris, Chesney, Devine, Fullilove, *et al.* 1998; Kalichman and Nachimson 1999; Wolitski, Rietmeijer, Goldbaum & Wilson 1998).

Stigma and discrimination have taken their toll in Ethiopia, not only in the personal (i.e. family, relationships and housing) and social (i.e. work place, schools) areas, but also within the medical services, discouraging people from being tested for HIV, receive care and other-related services (Kloos 2007). In the country, hospital bed occupancy rate due to HIV-related illness or AIDS-related illness has reached over 50% in urban hospitals, further stressing the already overburdened health system creating a severe burden to the health service system (Tibebu, G/Mariam & Belachew 2007). Home-based care would thus offer a feasible alternative to hospital-based care by mobilizing the latent reservoir of human resources and in alleviating some of the stigma and discrimination that exist within a community (Tibebu *et al.* 2007). Stigma and discrimination are key barriers in the delivery of care to PLHIV (Constance A Carrino 2005). HIV-related stigma and discrimination have fuelled the transmission of HIV, creating major barriers to prevent further infection and control of the epidemic, to alleviate its' impact and for the provision of adequate care, support and treatment services (UNAIDS 2005). Threat appeals, persuasive messages that evoke some level of fear, have been used successfully to disseminate various types of information to the general public (Gore and Bracken 2005). The Extended Parallel Process Model (EPPM) is one of the commonly used theories to aid in the planning of social marketing programs (Witte 1992). EPPM focuses on emotional responses that will impact on an individual's or group's motivation and behavior (Glanz, Rimer & Lewis 2002). The EPPM utilizes the protection motivation theory linkages among perceived levels of severity, susceptibility, response efficacy and self-efficacy that lead to acceptance and, ultimately, attitude, intention and behavior changes (Murray-Johnson, Witte, Patel, Orrego, Zuckerman, Maxfield, *et al.* 2004). The model focuses on messages that are received individually and collectively. The EPPM model was first developed to explain individual behavior; it has since been directly applied to the analysis of collective behavior (Barnett, Balicer, Thompson, Storey, Omer, Semon, *et al.* 2009). The EPPM is a model of health behavior change that integrates nearly 50 years of research and theorizing, it has been used across a wide variety of topics (e.g. HIV/AIDS, cancer, occupational safety, environmental risks) and populations and races (e.g. Hispanics, Kenyans, juvenile delinquents, the elderly, etc.) with good empirical basis (Leventhal 1970, 1971; Leventhal, Safer & Panagis 1983). For this study, EPPM was selected given that it is most appropriate for motivational assessment (as opposed to awareness or knowledge) campaigns, where the focal audience already has knowledge about HIV/AIDS, PLHIV and stigma, and discrimination. A high level of knowledge was reported by Ethiopia Demographic Health Survey and Behavioral Surveillance Survey respondents as well as found in other surveys in the country (CSA 2005; Ministry of Health/HIV/AIDS Prevention and Control Office: Ethiopia 2002). This study was designed to examine the psychosocial impact of stigma and discrimination and its effect on care seeking behavior of PLHIV in Southern Nations, Nationalities and People's Region (SNNPR) Ethiopia through the application of the EPPM.

## Methods

### Study setting and context

The study was conducted in Arba Minch town and nearby areas in SNNPR, Ethiopia. This study was conducted from November 2008 to January 2009 using qualitative methods, which enabled us a better approach to meet our objective. In-depth interviews (IDIs) with purposively selected PLWHIV and key informants were conducted. Landlords, work place representatives, community counselors, home-based care givers and health professionals (usually VCT counselors) and others in the town and nearby areas were chosen in order to attain in-depth understanding of the relevant issues. Focus group discussions (FGDs) were held with PLHIV to complement the information obtained from the key informants. The sample size for this study was not decided a priori, as it was dependent on the degree to which the incoming data answered the objective of the study. We were guided on the basis of information saturation. Whenever the information that was being analyzed became redundant, the research team made the decision to stop any additional interviews from being conducted.

### Participants' recruitment and selection

Saturation and redundancy of information, though indeterminate measures, were used to limit the questions as well as the number of key informants interviews and FGDs. Our aim was to collect information on a wide range of experiences, perspectives and behaviors. Heterogeneous sampling technique was used to get key informants from different areas (urban and rural), positions, associations, gender and social groups. Initially, associations of PLHIV in the town were identified and their representatives were contacted, explained the purpose of the study and asked to identify, among their members, those who were expressive and knowledgeable about issues relevant to our study. Snowball sampling technique was also used to identify additional key informants. These informants were recruited from their work place; home and other places were also included. The participants, who came to the study site through recruitment generated through the snowball sampling, were included. Questions were asked considering the informant's age, sex, marital status and social standing. This purposive sampling method was carefully used to select key informants that characterize or shed light on the study question based on the availability for enrollment.

### Data collection procedure

IDIs and FGDs were the methods of data collection used in this study and conducted by trained interviewers. IDIs were one-to-one and interactive; study participants were encouraged to take an active role in establishing the flow of the interview. Open-ended questions were used to collect relevant information. Questions were tailored to each informant's characteristics regarding age, gender, marital status and social standing. Questions that reached saturation were removed every evening after transcribing the day's work and doing preliminary analysis. New questions were added whenever an information gap was identified. The questions included knowledge, experience and opinion about HIV/AIDS services including stigma and discrimination. Attempts were also made to conduct a second interview with key informants, as it was deemed necessary to clarify some points identified during data analysis, to ask the reasons for their claims as well as to look for deeper explanation. Data were collected by two team researchers with qualitative data collection experience. The interviews were tape recorded. A total of 19 IDIs with key informants and four FGDs were conducted.

### Data analysis

Preliminary manual analysis was an inherent part of the data collection. As more data were collected, the meaning of certain ideas and concepts evolved into a concept matrix, which also helped in the revision and refinement of the questions as the study proceeded. All authors read the transcripts independently and developed a coding frame for the analysis. The first and second authors coded all the transcripts, and all the authors, except H. R. E., independently read the material and contributed in negotiating the final categories and their contents. Each audiotaped interview was transcribed in Amharic language and then translated to English. Thematic content analysis was performed on the data. Contact summaries were written in English for each interview. The contact summary reflected the output of each contact with respect to the themes that were formulated initially, the general output of the encounter and finally, the issues that were already saturated and those that needed further clarification. Exceptions and minority opinions were also identified and tracked to get improve our understanding.

The analysis primarily focused on collected data in the form of expanded field notes and transcripts of recorded interviews. Images and sounds such as facial expressions, promptness or reluctance in responding to questions, emphatic nature of the responses, and frustrations in addressing certain issues were also systematically interpreted and their meanings noted on paper to be incorporated into the analysis. Data reduction was performed to get the overall sense of the data collected, to distinguish central and secondary themes and to separate the essential from the non-essential. To understand how the key domains were organized into a framework, we examined quotes and discussions to illustrate each theme and to describe the effect of stigma and discrimination.

Ethical clearance was obtained from institutional review board of Arba Minch College of Health Sciences (AMCHS) ethical clearance committee. Written permission was obtained from the selected government, non-government organization (NGO) and association's offices. Verbal consent was obtained from each participant prior to data collection. Privacy and confidentiality were maintained throughout the study.

## Results

### Characteristics of the study population

We conducted 19 IDIs and 4 FGDs at Arba Minch town and nearby Kebeles (the smallest administrative unit/village in Ethiopia) with PLHIV and other key informants. Out of the 19 IDIs, 4 were conducted with PLHIV, 4 with neighbors of the PLHIV, 2 with community counselors, 2 with home-based care givers, 4 with health professionals and 3 with other individuals who were not part of any of the latter groups. A total of 19 key informants, 12 men and 7 women, with an age that ranged from 20 to 50 years were recruited. The FGDs were held with PLHIV from different associations; 3 from Arba Minch town representing urban population and another 1 with PLHIV from Shelle Kebele representing the rural population. A total of 32 people participated in the FGD (8 participants/group). Most of the key informants were married, while the FGD participants were divorced and almost all key informants and FGD participants have completed at least the primary level of education.

### Perceptions about the experience of stigma and discrimination on care seeking behavior

Each of the dimensions of the EPPM theoretical framework was evaluated (i.e. susceptibility, severity, self-efficacy and response efficacy) through experience of stigma and discrimination

### Perceived susceptibility to stigma and discrimination

According to the PLHIV participants, the magnitude of stigma and discrimination has been reduced when compared to previous years. It is, however, changing its nature from discernible physical stigma to a being concealed, this being affected by various factors, including: serostatus disclosure, rural residence and level of awareness about the disease and its transmission. A high proportion of key informants and 24 of the FGD participants agreed that PLHIV who had not disclosed are more susceptible to stigma and discrimination than those who have disclosed their serostatus.

A middle-aged woman living with HIV from Arba Minch responded to the question asked about the effect of not disclosing her serostatus on stigma and discrimination by saying:

Before disclosing my result to the public, peoples suspected that I could infect any of them as revenge; thus, some of them were telling their children not to take any food from me due to a belief that I may mix blood with it.

A 35-year-old married female with four daughters explained her approach to a PLHIV who already had disclosed their serostatus to the public as:

Being aware, at the start (before he disclosed his result), we were curious about all his activities for fearing deliberate transmission of the disease; later he disclosed his serostatus to the public letting us change our attitude towards him; Now, we are compassionate towards him. I just see him as one of my kin; we share what we have together without any hesitation …

Place of residence was also an another factor associated with stigma and discrimination of PLHIV. Key informants from rural areas reported being highly susceptible to stigma and discrimination when compared to reports from urban informants.

A 23-year-old student who is a neighbor of female PLHIV in rural Kebele explained:

My neighbor has been isolated for too long from the community as people are not free to join her. The main reason is that rural people are hardly aware about HIV/AIDS and its transmission; moreover, in our culture such diseases are highly stigmatized.

Pertaining to the factors responsible for these problems, most of the key informants and some of the FGD participants blamed the information contained in health messages that have been disseminated through the mass media as a leading factor for the prevailing stigma and discrimination followed by the poor awareness of the community about the disease and its transmission. Some of the key informants also blamed the government and religious institutions for not taking their part efficiently as would have been expected from them.

### Perceived severity to stigma and discrimination

Almost all of the key informants mentioned that stigma and discrimination affect self-esteem of PLHIV, which resulted in stress that in turn leads to mental health and other health-related problems. Gastric ulcer was reported as being one of the most common health issues; this represents an additional burden for those on highly active antiretroviral therapy. Another effect related to stigma and discrimination is reflected on the care seeking behavior of PLHIV. According to participants' responses, those PLHIV who had not disclosed their result were considerably less likely to utilize the care and support rendered to them by different governmental and non-governmental organizations as compared to those who have.

A 42-year-old male living with HIV from a rural Kebele said:

I used to know many PLHIV who were afraid to disclose disease and to join PLHIV associations; these people hide themselves even during medical visits. One day, a workmate living with HIV who was not willing to disclose his result, asked me to call him for ART treatment while he was sitting distant from the clinic; furthermore, this man takes his ART medications shyly or may not take it at all if there are people around. For me, this inevitably contributes for non-adherence, thus facilitating drug resistance.

In addition to limited medical services, non-disclosing PLHIV are less likely to get socio-economic, psychosocial, legal or spiritual supports. On the contrary, those PLHIV who disclosed their result are more likely to accept different care and support services provided by different institutions.

A large proportion of the key informants and some of the FGD participants believed that the level of stigma and discrimination becomes tolerable as time goes on and after joining an association for PLHIV.

A middle-aged widowed woman reported that living with HIV:

Now (especially after joining this association) I don't feel much stigma and discrimination as what I was feeling four years ago when I learned my serostatus. Peoples' attitude towards PLHIV has improved.

On the other hand, PLHIV from rural areas are still frustrated by stigma and discrimination within rural communities.

A female PLHIV from rural Kebele expressed the situation despondently by saying:

I am severely affected by the stigma and discrimination from the people of my area; I escaped from there (rural Kebele) and came here (semi-urban Kebele). Unfortunately for me, I couldn't afford the living expenses of the urban life. Nowadays, I wish to better die than suffer this.

### Perception of response efficacy to stigma and discrimination

Getting care and support, mainly medical, socio-economic and legal, are an effective response to stigma and discrimination. Almost all FGD participants and key informants supported this observation.

A middle-aged woman living with HIV, who participated in an FGD said:

If I could get adequate economic and medical support, definitely I will feel comfortable despite the stigma and discrimination outside, as this can compensate the moral damage envisaged to my life.

Pertaining to the participants' preferences for care giving organizations/agents, most of the participants from both groups favored local NGOs followed by government's justice system as best care givers, while social facilities like Idir and Ekub, as well as religious leaders were not favored by them since they did not adequately carry out their responsibilities to alleviate the problems to the level that was expected. On the contrary, most key informants claimed the latter groups to be effective for raising community awareness and instituting favorable attitude toward PLHIV within the community.

### Perception of self-efficacy to stigma and discrimination

Only few of the FGD participants agreed to go to court for discernible or concealed type of stigma and discrimination, since the bureaucracy of these institutions and the socio-economic condition do not allow them to reach these facilities. On the other hand, almost all of the PLHIV key informants and FGD participants agreed that they were willing to utilize medical, spiritual, socio-economic and other supports to improve their quality of life. However, non-disclosing PLHIV may not have access to these services.

Nowadays, as was mentioned by the FGD participants, among the most important actions that should be taken to improve the quality of care and support, is the reduction of the prevailing stigma and discrimination toward PLHIV. In addition, devising a way for improving disclosure of serologic status was also emphasized.

## Discussion

A difference in the magnitude of stigma and discrimination was reported by residence, educational level (community awareness about the disease) and disclosure status. The current study has identified that the reported stigma and discrimination have decreased and appears to be more efficiently dealt with by PLHIV who have disclosed their status.

The level of stigma and discrimination appears to be higher for PLHIV who live in rural areas, within an uneducated community and to those who have not yet disclosed their serostatus. This is because communities with the low socio-demographic characteristics do exhibit the same for other conditions too (Leventhal *et al.* 1983). The higher the level of stigma and discrimination, the lesser the rate of disclosure; the lesser PLHIV utilize care and support services, the poorer the quality of life of PLHIV. Heckman, Somlai, Kalichman, Franzoi & Kelly (1998) found that rural people living with HIV reported significantly lower satisfaction with life, more community stigma and heightened personal fear. It has also been described that stigmatized and discriminated PLHIV skipped doses of medication to avoid the stigma associated with publicly taking medication that could disclose their HIV status (Golin *et al.* 2002). Fear of being identified as having HIV or AIDS may discourage a person from getting tested, from accessing medical services and medications, and from disclosing their HIV status to family and friends (Sayles, Ryan, Silver, Sarkisian & Cunningham 2007). Fear, stigma and discrimination have continued to accompany the HIV pandemic (UNAIDS 2000). Actions to reduce or protect against discrimination and stigma may be one of the most significant steps that can be taken toward improving the psychosocial wellbeing of PLHIV (Sadoh, Sadoh, Fawole, Oladimeji & Sotiloye 2009). This might be mainly due to the fact that higher perceived susceptibility and perceived severity to stigma and discrimination leads to decreased motivation, which in turn results in reduced performance and productivity.

Stigma and discrimination have changed to a more complicated and concealed type, which can severely affect PLHIV's psychological well-being unless the proper actions are taken. Similar results have been reported in other studies (Leventhal 1971; Tibebu *et al.* 2007). Although stigma and discrimination were a concern for almost all PLHIV, it was perceived to severely affect the life of rural and non-disclosing PLHIV more. Stigma and discrimination are associated with negative health outcomes for people living with HIV including, greater severity of AIDS-related symptoms, lower perceived general health, and less health care satisfaction (Bird, Bogart & Delahanty 2004). The stigma related to HIV/AIDS and the behaviors associated with HIV risk have resulted in significant barriers to disclosing one's HIV serostatus (Kalichman and Nachimson 1999). In a study in southwest Ethiopia, it was reported that 79.4% individuals did not want to disclose their HIV-positive serostatus due to fear of stigma and discrimination (Deribe, Woldemichael, Wondafrash, Haile & Amberbir 2008). Interventions to reduce stigma both at the community level (e.g. education and media messages) and at the individual level (peer counseling) may be beneficial (Lam, Naar-King & Wright 2007).

Some of the limitations that we identified in our study were the lack of external validity, as we used purposeful sampling strategies and one health education model/theory may not explain all factors that determine behavioral change. In addition, this study examines only proximal factors for risk communication variables. Whereas the study used an empirically tested model to guide the assessment of the constructs, thus the findings of the study are reliable. Qualitative research also provides an approach that captures a wide diversity of ideas and practices which likely would not have been detected using a quantitative approach. The identification of stigma and discrimination questions was selected systematically from peer-reviewed literature and reports. In addition, the participants included in our study were heterogeneous, thus representing a broad variety of groups within the Ethiopian society.

## Conclusions and implications for practices

Our findings suggest that the experience of stigma and discrimination has an implication on care and support seeking behavior in the study area. Progress has been reported in the way of alleviating stigma and discrimination and also in the way the population expresses it. Though the nature of stigma and discrimination is changing from a discernible to a more concealed and complicated type and affected by different factors, it is possible to remark that the problem has come to a little concern for many PLHIV. The messages that were initially disseminated through mass media (e.g. radio and television) combined with limited effort to raise awareness within the community about the disease were identified as factors that affect the current level of stigma and discrimination. Associations of PLHIV were found to be helpful regarding disclosure.

Based on our findings, it would be important to expand them by studies that use a mixed qualitative and quantitative approach and that are able to capture a larger sample in other regions in Ethiopia with the aim of making the results generalizable to the wider populations with the objective of providing the necessary information to develop programs that address stigma and discrimination using a context sensitive approach. Finally, the findings are significant to the setting with generalized HIV epidemics, especially to cultural settings similar to Ethiopia.
